# Association of Insurance Type With Colorectal Surgery Outcomes and Costs at a Safety-Net Hospital: A Retrospective Observational Study

**DOI:** 10.1097/AS9.0000000000000215

**Published:** 2022-11-07

**Authors:** Jasmine C. Tetley, Michael A. Jacobs, Jeongsoo Kim, Susanne Schmidt, Bradley B. Brimhall, Virginia Mika, Chen-Pin Wang, Laura S. Manuel, Paul Damien, Paula K. Shireman

**Affiliations:** From the * Department of Surgery, University of Texas Health San Antonio, San Antonio, TX; † Department of Population Health Sciences, University of Texas Health San Antonio, San Antonio, TX; ‡ Department of Pathology and Laboratory Medicine, University of Texas Health San Antonio, San Antonio, TX; § University Health, San Antonio, TX; ∥ Department of Information, Risk, and Operations Management, Red McCombs School of Business, University of Texas, Austin, TX; ¶ Departments of Primary Care & Rural Medicine and Medical Physiology, School of Medicine, Texas A&M Health, Bryan, TX.

**Keywords:** colorectal surgery, insurance status, social risk factors, preoperative acute serious conditions, textbook outcomes, variable costs

## Abstract

Association of insurance type with colorectal surgical complications, textbook outcomes (TO), and cost in a safety-net hospital (SNH).

**Background:**

SNHs have higher surgical complications and costs compared to low-burden hospitals. How does presentation acuity and insurance type influence colorectal surgical outcomes?

**Methods:**

Retrospective cohort study using single-site National Surgical Quality Improvement Program (2013–2019) with cost data and risk-adjusted by frailty, preoperative serious acute conditions (PASC), case status and open versus laparoscopic to evaluate 30-day reoperations, any complication, Clavien-Dindo IV (CDIV) complications, TO, and hospitalization variable costs.

**Results:**

Cases (Private 252; Medicare 207; Medicaid/Uninsured 619) with patient mean age 55.2 years (SD = 13.4) and 53.1% male. Adjusting for frailty, open abdomen, and urgent/emergent cases, Medicaid/Uninsured patients had higher odds of presenting with PASC (adjusted odds ratio [aOR] = 2.02, 95% confidence interval [CI] = 1.22–3.52, *P* = 0.009) versus Private. Medicaid/Uninsured (aOR = 1.80, 95% CI = 1.28–2.55, *P* < 0.001) patients were more likely to undergo urgent/emergent surgeries compared to Private. Medicare patients had increased odds of any and CDIV complications while Medicaid/Uninsured had increased odds of any complication, emergency department or observations stays, and readmissions versus Private. Medicare (aOR = 0.51, 95% CI = 0.33–0.88, *P* = 0.003) and Medicaid/Uninsured (aOR = 0.43, 95% CI = 0.30–0.60, *P* < 0.001) patients had lower odds of achieving TO versus Private. Variable cost %change increased in Medicaid/Uninsured patients to 13.94% (*P* = 0.005) versus Private but was similar after adjusting for case status. Urgent/emergent cases (43.23%, *P* < 0.001) and any complication (78.34%, *P* < 0.001) increased %change hospitalization costs.

**Conclusions:**

Decreasing the incidence of urgent/emergent colorectal surgeries, possibly by improving access to care, could have a greater impact on improving clinical outcomes and decreasing costs, especially in Medicaid/Uninsured insurance type patients.

## Abstract 2

Mini-Abstract A retrospective cohort study (1078 cases) assessing the association of insurance type (Private, Medicare, Medicaid/uninsured) with complications and costs adjusted for frailty, open versus laparoscopic approach, and case status. Markedly increased rates of urgent/emergent surgeries in Medicaid/uninsured patients drive the increased odds of complications and costs compared to Private patients.

## INTRODUCTION

Frailty^1^ and social risk factors^2^ significantly impact colorectal surgical outcomes; however, value-based medicine risk adjustment models fail to account for these factors. Additionally, the impact of frailty on patient outcomes is not captured using standard Hierarchical Condition Category (HCC) risk adjustment.^3,4^ High Social Vulnerability Index scores in colorectal surgery patients were associated with higher risk of postoperative complications and index hospitalization expenditures,^2^ but are not included in risk adjustment. Safety-net hospitals (SNH) serve higher proportions of low socioeconomic status (SES) patients with higher severity of illness scores, higher rates of emergency surgeries, and longer hospital length of stay (LOS).^5^ Hospital Readmission Reduction Program and other pay for performance (P4P) programs unintentionally contribute to widening disparities in healthcare and outcomes, penalizing SNH, and further limiting resources to treat vulnerable populations.^6–8^

High-burden, SNH have higher postoperative complications and costs compared to low-burden hospitals.^5,9,10^ Insurance status/type is a common proxy used for patient SES.^11–13^ Moreover, dual-eligibility for Medicare and Medicaid is indicative of poverty and plays a fundamental role in predicting surgical outcomes,^12,14^ along with urgent/emergent surgeries,^15^ frailty,^1,11,13,16–18^ and open compared to laparoscopic surgeries.^12^ Uninsured or Medicaid patients experience increased rates of emergency procedures, complications, and mortality.^14,19,20^ Additionally, the current NSQIP risk calculator underestimates the risk of complications for emergency surgeries^21^ and does not provide risk prediction for urgent cases.^15^ Failure to account for factors beyond clinicians’ control (eg, increased presentation acuity and social risk factors) in value-based medicine when assessing outcomes of high-burden facilities may continue to widen disparities in access, care, and outcomes in vulnerable populations.^6,18^

Textbook outcomes (TO) is a composite metric that has been increasingly used to assess surgical outcomes.^22,23^ Social risk factors may not consistently affect outcomes; therefore, including multiple outcomes of interest may more comprehensively assess the effects and costs of social risk factors, especially in underinsured/uninsured patients.

We assessed the association of Private, Medicare, and Medicaid/Uninsured (ie, Medicaid, dual-eligible Medicare/Medicaid, self-pay, indigent programs) health insurance type with complications and cost after colorectal surgeries in an SNH with a large range of SES patients. We hypothesize that after adjusting for frailty and open versus laparoscopic procedure type, increased presentation acuity, measured by presenting with acute serious conditions and urgent or emergent cases in patients with Medicaid/Uninsured insurance type will be associated with higher complications and index hospitalization costs compared to patients with Private insurance.

## MATERIALS AND METHODS

### Study Population and Data

This retrospective cohort study followed STROBE Reporting Guidelines^24^ and used local, identified data on all patients undergoing colorectal procedures present in the 2013-2019 American College of Surgeons National Surgical Quality Improvement Program (NSQIP) at a single facility, serving as an academic medical center and SNH. NSQIP registry was used for cohort identification. NSQIP provides standardized definitions of preoperative risk factors and complications.^25^ The Institutional Review Board of the University of Texas Health San Antonio approved this study.

### Patient Preoperative and Operative Variables

Frailty was measured using the recalibrated Risk Analysis Index (RAI)^26^ using preoperative variables from NSQIP as previously described.^16^ The RAI has been validated using both Veterans Affairs Quality Improvement Program and NSQIP datasets^26^ and exhibits collinearity with the Charlson Comorbidity Index.^1^ RAI has been used for risk adjustment for medical comorbidities in multiple studies.^16,17,27,28^ RAI scores were grouped into robust (≤20), normal (21–29), frail (30–39), and very frail (≥40).

Patients presenting with preoperative acute serious conditions (PASC) were defined using 6 NSQIP present at the time of surgery (PATOS) variables and NSQIP variables defining acute renal failure (with or without dialysis required) within 2 weeks before surgery, as previously described (Supplemental Table 1, http://links.lww.com/AOSO/A177 lists the NSQIP variable names).^16^ Case status was determined from NSQIP variables with urgent cases being defined as neither elective nor emergency, as determined by ”no” responses to the ELECTSURG and EMERGNCY variables.^15^ Procedures were categorized as open or laparoscopic surgeries using NSQIP principal Current Procedural Terminology Codes (Supplemental Table 2, http://links.lww.com/AOSO/A177).

### Any and Clavien-Dindo IV 30-Day Complications

Clavien-Dindo classifies complications based on their treatments.^29^ We approximated Clavien-Dindo IV (CDIV) complications using the NSQIP variables of postoperative septic shock, postoperative dialysis, pulmonary embolus, myocardial infarction, cardiac arrest, prolonged ventilation, reintubation, or stroke as previously reported.^16^ Unplanned reoperations were defined using the NSQIP variable REOPERATION1 as present or absent. Any complication was defined using the CDIV and reoperation NSQIP variables plus an additional 11 NSQIP variables defining postoperative complications.

### TO Composite Variable

TOs were defined as surgeries with the absence of 30-day CDIV complications, unplanned reoperations, 30-day mortality after the date of surgery, and 30-day after the date of discharge from the index hospitalization readmissions and emergency department or observations stays (EDOS).

### Mortality

Mortality was defined as death within 30 days of the index colorectal surgical procedure. Dates of death were obtained from ACS NSQIP and cross referenced from our data warehouse using the electronic health records of the local health system as well as the Social Security Death Master File.

### Insurance Type and Cost Data

The identified, local NSQIP data were merged with electronic health records and managerial accounting data to determine insurance type and the variable cost of the index hospitalization. Insurance type was categorized based upon billing data for the encounter supplemented by EHR data and defined as (1) Private including TRICARE and workers compensation; (2) Medicare; and (3) Medicaid/Uninsured including Medicaid, dual enrollment in Medicare/Medicaid, charity care, self-pay with ≤1% of charges paid, or county indigent care programs (Supplemental Table 3, http://links.lww.com/AOSO/A177). “Other” included encounters billed to the Veterans Administration, Department of Corrections, or self-pay with >1% of charges collected and were excluded (n = 13).

We defined variable costs as related directly to patient care occurring during the encounter, such as supplies/salaries and include direct variable costs that vary directly with the quantity of resources provided for patient care.^30,31^ Direct variable costs are accounted primarily using direct measurements from a bottom-up approach rather than calculated estimates derived from charges. Hospital fixed costs, outpatient and professional fees were not included. We used variable costs, as fixed costs are not directly related to patient care and vary between hospitals.^31^ The natural logarithm of variable costs was used, as previously described^30^ after adjusting costs to 2019 dollars using the Personal Health Care Index.^32^

### Management of Missing Variables

Cases were excluded due to (1) perineal and transsacral only procedures; (2) missing or inaccurate cost variables; and (3) “Other” insurance type.

### Study Outcomes

Clinical outcomes of interest were 30-day unplanned reoperations, any complication, severe/life-threatening CDIV complications, readmissions, EDOS, TO composite variable, and variable costs for the index surgery hospitalization adjusted for RAI, case status, open versus laparoscopic procedure, and insurance type.

### Statistical Analysis

Categorical data were summarized using count and percentage and continuous data using mean and standard deviation (SD). Chi-square tests and F-tests were used to test for difference between groups for categorical and continuous variable. Logistic regression analyses were performed for (1) PASC and case status adjusting for RAI, open abdominal procedures, and insurance types; (2) open abdominal procedures adjusting for RAI, insurance type, PASC and case status; (3) complications, EDOS, readmissions and TO adjusting for RAI, open abdominal procedures, insurance type, and case status; and (4) TO subgroup analyses for elective and urgent/emergent cases. Natural logarithms were used to normalize the skewed LOS and variable costs for the index hospitalization, which reduces the impact of extreme values, as previously described.^30,33^ Percent change/relative difference was calculated using the exponential function; %change = (*e*^estimated coefficients^ − 1) × 100. Analyses were performed using R version 4.1.0 (2021-05-18).

## RESULTS

### Population Characteristics

**FIGURE 1.**
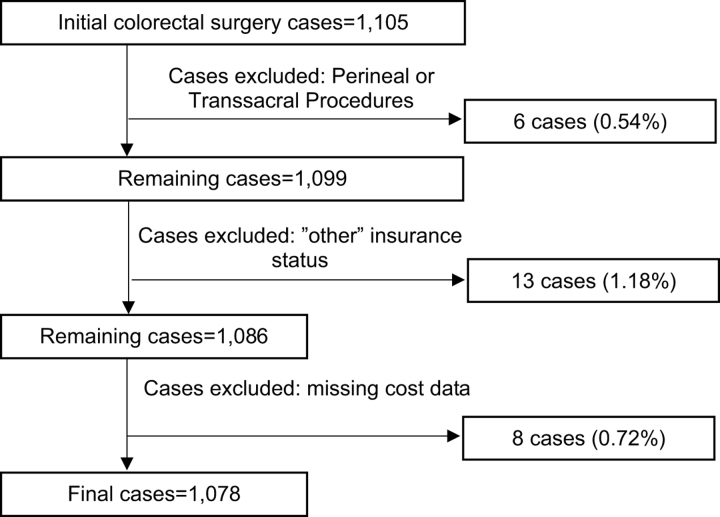
Flow diagram of study cohort. National Surgery Quality Improvement Program inpatient cases from 2013 to 2019. Cases were excluded for perineal or transsacral only procedures, “other” insurance status, and missing cost data. “Other” insurance status was defined as encounters billed to the Veterans Administration, Department of Corrections, or self-pay with >1% of charges collected.

Our cohort consisted of 1,078 cases of inpatient procedures at a major urban SNH (Fig. 1). Cases (Table 1) were more commonly performed on males (53.1%) and White patients (90.4%) with the majority identifying as Hispanic ethnicity (64.7%). Most cases were performed on patients with Medicaid/Uninsured insurance type (57.4%), followed by Private (23.4%) and Medicare (19.2%). Most cases were performed on robust (60.3%) and normal (27.5%) patients based on RAI scores. Only 9.7% and 2.5% of patients were frail and very frail, respectively, with Medicare patients exhibiting higher rates of frailty. Complication rates were higher in Medicare and Medicaid/Uninsured patients compared to Private.

**TABLE 1.** Patient Characteristics and Clinical Outcomes by Insurance Type

	Overall	Private	Medicare	Medicaid/Uninsured	*P*
Number (%)*	1078	252 (23.4)	207 (19.2)	619 (57.4)	
Age mean (SD)	55.2 (13.4)	51.43 (11.2)	68.37 (10.0) (11.0)	52.33 (12.5)	**<0.001**
Sex (male)	572 (53.1)	132 (52.4)	108 (52.2)	332 (53.6)	0.908
Race					0.329
White	974 (90.4)	223 (88.5)	194 (93.7)	557 (90.0)	
Black	74 (6.9)	20 (7.9)	8 (3.9)	46 (7.4)	
Other	21 (1.9)	7 (2.8)	2 (1.0)	12 (1.9)	
Missing	9 (0.8)	2 (0.8)	3 (1.4)	4 (0.6)	
Hispanic Ethnicity	698 (64.7)	119 (47.2)	112 (54.1)	467 (75.4)	**<0.001**
RAI					**<0.001**
Robust (≤20)	650 (60.3)	192 (76.2)	45 (21.7)	413 (66.7)	
Normal (21-29)	296 (27.5)	46 (18.3)	110 (53.1)	140 (22.6)	
Frail (30-39)	105 (9.7)	11 (4.4)	42 (20.3)	52 (8.4)	
Very Frail (≥40)	27 (2.5)	3 (1.2)	10 (4.8)	14 (2.3)	
PASC	149 (13.8)	19 (7.5)	32 (15.5)	98 (15.8)	**0.004**
Laparoscopic	431 (40.0)	116 (46.0)	90 (43.5)	225 (36.3)	**0.016**
Open Abdomen	647 (60.0)	136 (54.0)	117 (56.5)	394 (63.7)	**0.016**
Case Status					**<0.001**
Elective	620 (57.5)	175 (69.4)	129 (62.3)	316 (51.1)	
Urgent	340 (31.5)	61 (24.2)	50 (24.2)	229 (37.0)	
Emergent	118 (10.9)	16 (6.3)	28 (13.5)	74 (12.0)	
LOS median (Q1-Q3)					
Q1 (25th percentile)	4	4	4	5	
Median (50th percentile)	7	5	7	7	
Q3 (75th percentile)	13	8.25	15	13	
IQR (Q3–Q1)	9	4.25	11	8	
LOS mean (SD)	10.5 (11.5)	8.7 (11.2)	11.0 (10.5)	11.1 (11.8)	**0.015**
Complications					
Any	470 (43.6)	84 (33.3)	103 (49.8)	283 (45.7)	**0.001**
CDIV	113 (10.5)	13 (5.2)	36 (17.4)	64 (10.3)	**<0.001**
Reoperation	88 (8.2)	16 (6.3)	20 (9.7)	52 (8.4)	0.412
30-day Mortality	26 (2.4)	3 (1.2)	8 (3.9)	15 (2.4)	0.178
30-day EDOS	133 (12.3)	11 (4.4)	17 (8.2)	105 (17.0)	**<0.001**
30-day Readmission	223 (20.7)	37 (14.7)	40 (19.3)	146 (23.6)	**0.011**
Textbook Outcomes	669 (62.1)	195 (77.4)	123 (59.4)	351 (56.7)	**<0.001**

30-day EDOS and readmission defined as 30 days from date of discharge from index hospitalization.

30-day mortality defined as 30 days from the date of index surgery.

*Percent calculation by row, the rest of the percent calculations were by column.

IQR indicates interquartile range; Q, quartile.

Bolded *P*-values are significant at the <0.05 level.

### Increased PASC in Medicaid/Uninsured Patients and Increased Urgent/Emergent Cases in Medicaid/Uninsured and Medicare Patients

Rates of patients presenting with PASC (13.8%) were highest in Medicaid/Uninsured (15.8%) and Medicare (15.5%) patients versus Private (7.5%, *P* = 0.004, Table 1). Medicaid/Uninsured patients had higher odds of presenting with PASC (adjusted odds ratio [aOR] = 2.02, 95% confidence interval [CI] = 1.22–3.52, *P =* 0.009; Table 2) compared to Private after adjusting for frailty and open procedures. Odds of undergoing an urgent/emergent case were lowest in robust compared to normal patients and higher for patients undergoing open abdomen surgeries compared to laparoscopic (aOR = 2.26, 95% CI = 1.70–3.03, *P <* 0.001) and Medicaid/Uninsured patients (aOR = 1.8, 95% CI = 1.28–2.55, *P <* 0.001) compared to Private. Patients presenting with PASC had higher odds of undergoing urgent/emergent surgeries (aOR = 26.65, 95% CI = 13.61–60.28, *P* < 0.001). All 3 insurance types had similar odds of undergoing open abdominal surgeries after adjusting for frailty, PASC, and urgent/emergent procedures.

**TABLE 2.** PASC, Urgent/Emergent Case Status and Open Abdominal Procedures Adjusted Odds Ratios

	PASC			Urgent/Emergent Cases			Open Abdomen		
	aOR	CI	*P*	aOR	CI	*P*	aOR	CI	*P*
RAI (Ref = Normal 21–29)									
Robust (≤20)	0.79	0.51–1.23	0.291	0.62	0.44–0.86	**0.005**	0.89	0.65–1.23	0.490
Frail (30–39)	1.15	0.62–2.06	0.649	1.36	0.82–2.26	0.235	2.33	1.38–4.05	**0.002**
Very frail (≥40)	3.23	1.37–7.53	**0.007**	1.71	0.61–5.07	0.312	6.08	1.68–39.12	**0.018**
Open abdomen (Ref = Laparoscopic)	5.23	3.20–9.03	**<0.001**	2.26	1.70–3.03	**<0.001**			
Insurance (Ref = Private)									
Medicare	1.81	0.95–3.52	0.075	0.79	0.49–1.28	0.344	0.78	0.51–1.19	0.248
Medicaid/Uninsured	2.02	1.22–3.52	**0.009**	1.80	1.28–2.55	**<0.001**	1.17	0.85–1.60	0.331
PASC				26.65	13.61–60.28	**<0.001**	3.23	1.91–5.73	**<0.001**
Urgent/emergent (Ref = Elective)							2.25	1.68–3.01	**<0.001**

Ref indicates reference value.

Bolded *P*-values are significant at the <0.05 level.

PASC variable distribution (Supplemental Table 1, http://links.lww.com/AOSO/A177) was similar between insurance types, except for sepsis which was lowest in Medicare patients. Rates of urgent/emergent cases were highest in the Medicare (100.0%) and Medicaid/Uninsured (94.9%) patients presenting with PASC.

### Distribution of CDIV Complications and Increased Odds of 30 Days From Date of Surgery Complications in Medicare and Medicaid/Uninsured Patients

Rates of CDIV complications were 10.5% overall and were higher in Medicare and Medicaid/Uninsured patients (17.4% and 10.3%, respectively, *P* < 0.001; Table 1) compared to Private (5.2%). Any complication (43.6%) was also higher in Medicare and Medicaid/Uninsured patients (49.8% and 45.7%, respectively, *P* = 0.001). The distribution of CDIV complications (Supplemental Table 1, http://links.lww.com/AOSO/A177) was similar between insurance types except myocardial infarction was highest in Medicare patients (25.0%) and Private (23.1%) compared to Medicaid/Uninsured (7.8%, *P* = 0.048).

Medicare patients had higher odds of any complication (aOR = 1.57, 95% CI = 1.02–2.42, *P* = 0.042; Table 3) and CDIV complications (aOR = 2.56, 95% CI = 1.26–5.46, *P* = 0.011), while Medicaid/Uninsured patients had higher odds of any complication (aOR = 1.41, 95% CI = 1.02–1.96, *P* = 0.040) versus Private.

**TABLE 3.** Any Complication and Outcome Variables Used in Textbook Outcomes Adjusted for Frailty, Open Abdomen Procedures, Insurance Type and Urgent/Emergent Case Status

	Any Complication			CDIV Complications			Reoperations		
	aOR	CI	*P*	aOR	CI	*P*	aOR	CI	*P*
RAI (Ref = Normal 21–29)									
Robust (≤20)	0.75	0.55–1.03	0.074	0.45	0.27–0.75	**0.002**	1.15	0.67–2.04	0.610
Frail (30–39)	1.57	0.98–2.56	0.065	1.05	0.56–1.91	0.878	1.31	0.61–2.72	0.472
Very frail (≥40)	3.18	1.23–9.88	**0.026**	1.93	0.77–4.67	0.151	0.74	0.11–2.73	0.692
Open abdomen									
(Ref = Laparoscopic)	3.43	2.59–4.55	**<0.001**	3.03	1.76–5.54	**<0.001**	2.02	1.22–3.45	**0.008**
Insurance (Ref = Private)									
Medicare	1.57	1.02–2.42	**0.042**	2.56	1.26–5.46	**0.011**	1.60	0.76–3.39	0.215
Medicaid/Uninsured	1.41	1.02–1.96	**0.040**	1.53	0.83–3.02	0.197	1.24	0.71–2.31	0.467
Urgent/emergent									
(Ref = Elective)	1.41	1.08–1.85	**0.013**	4.22	2.63–6.99	**<0.001**	1.17	0.74–1.86	0.498
	30-Day Mortality			30-Day EDOS			30-Day Readmissions		
	aOR	CI	*P*	aOR	CI	*P*	aOR	CI	*P*
RAI (Ref = Normal 21–29)									
Robust (≤20)	0.26	0.08–0.78	**0.020**	1.06	0.68–1.69	0.807	0.98	0.68–1.43	0.936
Frail (30–39)	1.34	0.44–3.78	0.591	1.07	0.52–2.11	0.842	1.15	0.66–1.94	0.620
Very frail (≥40)	3.79	1.06–12.24	**0.029**	1.33	0.37–3.84	0.627	1.88	0.78–4.31	0.144
Open abdomen									
(Ref = Laparoscopic)	2.61	0.86–11.33	0.132	1.34	0.90–2.01	0.159	2.30	1.63–3.27	**<0.001**
Insurance (Ref = Private)									
Medicare	1.49	0.39–7.25	0.582	2.01	0.90–4.66	0.093	1.30	0.76–2.21	0.333
Medicaid/Uninsured	1.33	0.42–5.93	0.662	4.64	2.54–9.34	**<0.001**	1.69	1.14–2.55	**0.011**
Urgent/emergent									
(Ref = Elective)	6.91	2.30–29.87	**0.002**	0.71	0.48–1.05	0.093	0.92	0.67–1.26	0.595

30-day EDOS and Readmissions defined as 30 days from date of discharge from the index hospitalization.

30-day Mortality defined as 30 days from date of index surgery.

Bolded *P*-values are significant at the <0.05 level.

Urgent/emergent surgeries greatly increased 30-day mortality odds (aOR = 6.91, 95% CI = 2.30–29.87, *P* = 0.002) compared to elective cases.

Subgroup analysis including only elective (Supplemental Table 4, http://links.lww.com/AOSO/A177) or urgent/emergent (Supplemental Table 5, http://links.lww.com/AOSO/A177) cases demonstrated that Medicare patients had higher aOR for CDIV complications for elective cases and any complication for the urgent/emergent subgroup compared to Private insurance patients. Medicaid/Uninsured patients had higher aOR only for any complication in the urgent/emergent subgroup compared to the Private insurance group.

### Increased Odds of 30 Days From Date of Discharge EDOS and Readmissions in Medicaid/Uninsured Patients

Medicaid/Uninsured patients had higher odds of EDOS occurring (aOR = 4.64, 95% CI = 2.54–9.34, *P* = 0.001; Table 3) compared to Private. Medicaid/Uninsured patients also had higher odds of hospital readmissions (aOR = 1.69, 95% CI = 1.14–2.55, *P* = 0.011). Subgroup analysis including only elective (Supplemental Table 4, http://links.lww.com/AOSO/A177) or urgent/emergent (Supplemental Table 5, http://links.lww.com/AOSO/A177) cases showed EDOS had a higher aOR for Medicaid/Uninsured versus Private insurance group in the elective and urgent/emergent subgroups.

### Decreased Odds of TO in Medicare and Medicaid/Uninsured Patients

Both Medicare (aOR = 0.51, 95% CI = 0.33–0.80, *P* = 0.003; Table 4) and Medicaid/Uninsured (aOR = 0.43, 95% CI = 0.30–0.60, *P* < 0.001) patients had decreased odds of achieving TO compared to Private. Subgroup analyses of elective and urgent/emergent case status also demonstrated that Medicare and Medicaid/Uninsured patients exhibited decreased odds of TO compared to Private. Rates of TO for Private, Medicare and Medicaid/Uninsured patients were 79.4%, 67.4%, and 62.0% for elective cases (*P* < 0.001) and 72.7%, 46.2%, and 51.2% for urgent/emergent cases (*P* = 0.001), respectively.

**TABLE 4.** TOs Adjusted for RAI, Open Abdomen, and Insurance Type With Cases Status Subgroup Analyses

	TO All Cases			TO Elective			TO Urgent/Emergent		
	aOR	CI	*P*	aOR	CI	*P*	aOR	CI	*P*
RAI (Ref = Normal 21–29)									
Robust(≤20)	1.22	0.89–1.67	0.210	0.91	0.58–1.42	0.683	1.72	1.09–2.72	**0.020**
Frail (30-39)	0.89	0.56–1.42	0.628	0.83	0.41–1.71	0.611	1.02	0.54–1.91	0.950
Very Frail (≥40)	0.16	0.04–0.43	**<0.001**	0.09	0.00–0.57	**0.031**	0.22	0.05–0.72	**0.023**
Open abdomen (Ref = Laparoscopic)	0.44	0.33–0.59	**<0.001**	0.45	0.31–0.64	**<0.001**	0.42	0.26–0.67	**<0.001**
Insurance (Ref = Private)									
Medicare	0.51	0.33–0.80	**0.003**	0.54	0.30–0.96	**0.037**	0.41	0.20–0.83	**0.015**
Medicaid/Uninsured	0.43	0.30–0.60	**<0.001**	0.44	0.28–0.68	**<0.001**	0.37	0.20–0.64	**<0.001**
Urgent/emergent (Ref = Elective)	0.78	0.60–1.02	0.074						

Bolded *P*-values are significant at the <0.05 level.

### Medicaid/Uninsured Patients Have Longer Index Hospitalizations but Not After Adjusting for Urgent/Emergent Cases

Medicaid/Uninsured (17.51%, *P* < 0.001, Supplemental Table 6, http://links.lww.com/AOSO/A177) patients had longer %change LOS compared to Private. However, after adjusting for urgent/emergent case status, LOS of Medicaid/Uninsured patients were similar to Private. Urgent/emergent cases resulted in a 70.42% change in LOS compared to elective cases (*P* < 0.001).

### Increased Hospitalization Variable Costs in Medicaid/Uninsured Patients Were Similar to Private After Adjusting for Urgent/Emergent Cases

Medicaid/Uninsured patients (13.94%, *P* = 0.005) had higher %changes in variable costs compared to Private (Table 5). However, after adjusting for urgent/emergent cases, Medicaid/Uninsured patients had similar variable costs compared to Private patients. Urgent/emergent case status resulted in a 43.23 %change compared to elective case status (*P* < 0.001). Adjusting for any complication or CDIV complications increased the %change by 78.34% and 153.07%, respectively (Table 6). After adjusting for either any complication or CDIV complications, both Medicare and Medicaid/Uninsured patients exhibited similar %change of variable costs compared to Private.

**TABLE 5.** Variable Costs Adjusted for RAI, Open Abdomen, and Insurance Type: Medicaid/Uninsured Similar to Private Insurance Type After Adjusting for Urgent/Emergent Case Status

	Log(Variable Costs)				Log(Variable Costs)			
	%change	Estimates	CI	*P*	%change	Estimates	CI	*P*
Intercept		9.12	9.01–9.24	**<0.001**		9.03	8.92–9.14	**<0.001**
RAI (Ref = Normal 21–29)								
Robust (≤20)	−15.66	−0.17	−0.26 to −0.08	**<0.001**	−12.42	−0.13	−0.22 to −0.05	**0.003**
Frail (30–39)	21.39	0.19	0.06–0.33	**0.006**	18.14	0.17	0.03–0.30	**0.014**
Very frail (≥40)	39.52	0.33	0.09–0.58	**0.007**	29.99	0.26	0.03–0.50	**0.028**
Open abdomen (Ref = Laparoscopic)	51.04	0.41	0.34–0.49	**<0.001**	38.57	0.33	0.25–0.40	**<0.001**
Insurance (Ref = Private)								
Medicare	11.27	0.11	−0.01 to 0.23	0.085	11.72	0.11	−0.01 to 0.23	0.063
Medicaid/Uninsured	13.94	0.13	0.04–0.22	**0.005**	8.13	0.08	−0.01 to 0.17	0.081
Urgent/emergent (Ref = Elective)					43.23	0.36	0.28–0.44	**<0.001**

% change is calculated with marginal change of Log(direct costs) for one unit of each variable change below:

(*e*^(intercept+estimated coefficients)^ − *e*^intercept^)/*e*^intercept^ × 100, which is equal to (*e*^estimated coefficients^ − 1) × 100.

Ref indicates reference value.

Bolded *P*-values are significant at the <0.05 level.

**TABLE 6.** Variable Costs Adjusted for RAI, Open Abdomen, Insurance Type, Urgent/Emergent Case Status, and Complications

	Log(Variable Costs)				Log(Variable Costs)			
	% change	Estimates	CI	*P*	% change	Estimates	CI	*P*
Intercept		8.92	8.82–9.02	**<0.001**		9.01	8.92–9.11	**<0.001**
RAI (Ref = Normal 21–29)								
Robust (≤20)	−9.19	−0.10	−0.17 to −0.02	**0.015**	−7.43	−0.08	−0.15 to 0.00	0.051
Frail (30–39)	11.16	0.11	−0.01 to 0.22	0.080	15.86	0.15	0.03–0.27	**0.015**
Very frail (≥40)	13.73	0.13	−0.08 to 0.34	0.229	11.61	0.11	−0.10 to 0.32	0.304
Open abdomen (Ref = Laparoscopic)	18.21	0.17	0.10–0.24	**<0.001**	30.10	0.26	0.20–0.33	**<0.001**
Insurance (Ref = Private)								
Medicare	5.89	0.06	−0.05 to 0.16	0.282	4.66	0.05	−0.06 to 0.15	0.391
Medicaid/Uninsured	3.80	0.04	−0.04 to 0.12	0.350	6.64	0.06	−0.01 to 0.14	0.107
Urgent/emergent (Ref = Elective)	37.07	0.32	0.25–0.38	**<0.001**	28.19	0.25	0.18–0.32	**<0.001**
Any complication	78.34	0.58	0.51–0.65	**<0.001**				
CDIV complications					153.07	0.93	0.82–1.04	**<0.001**

Ref indicates reference value

Bolded *P*-values are significant at the <0.05 level.

Subgroup analysis for patients without any postoperative complications showed that Medicaid/Uninsured patients had 8.84% higher variable costs compared to Private insurance patients but had similar costs after adjusting for urgent/emergent cases (Supplemental Table 7, http://links.lww.com/AOSO/A177). For the TO patient subgroup, Medicaid/Uninsured patients had 13.48% higher variable costs versus Private and 9.58% higher variable costs after adjusting for urgent/emergent cases (Supplemental Table 8, http://links.lww.com/AOSO/A177).

## DISCUSSION

Medicare and Medicaid/Uninsured patients had higher odds of 30-day complications with decreased odds of achieving TO compared to the Private group. Contributing to the worse outcomes in Medicaid/Uninsured patients were the increased odds of presenting with PASC (aOR = 2.02) and undergoing urgent/emergent surgeries (aOR = 1.80) versus Private. Presenting with PASC was associated with 94.6% rate and aOR = 26.65 of undergoing an urgent/emergent surgery. Consistent with our data, uninsured patients were 3.54 times more likely to undergo emergent colorectal surgeries.^19^ Urgent/emergent surgeries had higher odds of complications in this study and in prior publications for urgent^15^ and emergent^19,34,35^ procedures, suggesting that Medicaid/Uninsured patients present under worse condition than privately insured patients. Urgent cases usually occur after a failed trial of medical management in unplanned hospitalizations. Numerous studies stratify cases into elective and emergent without categorizing urgent case status^19,34,35^ or stating how urgent cases were classified.^19,34,35^ Urgent cases were more common than emergent cases in all three insurance groups and highest (37.0%) in the Medicaid/Uninsured group (Table 1). Combining urgent and elective cases may disproportionately increase complication rates of vulnerable patients that have higher rates of urgent surgeries.

Open procedures in our study and others had higher odds of complications,^36^ reoperations,^36^ costs,^37^ and lower odds of achieving TO compared to laparoscopic surgeries. Medicaid/Uninsured patients displayed similar odds of undergoing open procedures as Private after adjusting for PASC and urgent/emergent cases (Table 2). This suggests that the higher rates of open procedures in Medicaid/Uninsured patients were due to increased presentation acuity and need for urgent/emergent surgeries, as opposed to not providing the alternative of laparoscopic surgery.

Medicaid/Uninsured patients had higher odds of 30-day EDOS (aOR = 4.64) and readmissions (aOR = 1.69). Emergency departments often serve as the primary healthcare source for low-SES patients with limited access to care.^38^ The strongest predictor for preventable readmissions was patients undergoing urgent/emergent colorectal procedures.^39^ Higher odds of readmission were observed in our Medicaid/Uninsured group with longer LOS given their higher rates of emergent cases, consistent with previous studies.^12,19^ Adjusting for patient SES showed similar readmission odds after major surgery between SNH and non-SNH, leading the authors to conclude that differences in patient case mix of low-SES patients, not quality of care, were responsible for higher readmission rates at SNH.^7^

Medicare and Medicaid/Uninsured patients had decreased odds of achieving the composite TO measure, regardless of case status. We chose TO as our primary outcome because composite measures often provide more comprehensive assessments of surgical outcomes than single variables.^40–42^ Our data demonstrate the utility of this approach. While all component variables had increased aOR for Medicare and Medicaid/Uninsured patients compared to the Private insurance group (Table 3), only CDIV complications were significant for Medicare and 30-day EDOS and readmissions for Medicaid/Uninsured patients. Thus, identification of healthcare disparities may be improved by composite variables secondary to the additive effects of each component. Prior studies using TO in colorectal surgery have been limited to oncological procedures.^43,44^ Insurance type has consistently been recognized as an independent risk factor for worse surgical outcomes^12,14,20,45^ and higher costs.^30,45,46^ Consistent with our results, a study on diverticular disease demonstrated the association of Medicaid and Uninsured patients with complicated preoperative presentations and increased mortality.^20^

The patients with Private insurance treated in our SNH had a similar complication rate (33.3%) to the previously reported complication rate (32.8%) of privately insured cohorts treated in low-burden hospitals.^47^ This suggests that poor outcomes are not a result of lower quality of care in SNH but due to patient-level differences.^48^ While some groups have established SNH provide equitable care,^49,50^ high-burden SNH have been associated with increased risk of complications^9,10^ and costs.^5^ Many colorectal surgery studies assess surgical outcomes across multiple healthcare systems based on safety-net burden^9,12^ rather than within a healthcare system, which ultimately compares institutions with vastly different patient populations. The National Academy of Medicine recommends studying the effect of social risk factors within a hospital system, especially one that cares for a range of SES patients, to target factors that distinguish between high and low quality of care.^51^ This study is one of the first to assess colorectal surgery outcomes following these recommendations and includes factors influencing the cost of care for patients in these insurance groups.

The Medicaid/Uninsured group was associated with increased adjusted odds of longer index hospitalization LOS and higher variable costs, but both were similar to Private after adjusting for urgent/emergent cases. Major factors impacting the %change in variable costs (Table 6) were urgent/emergent cases (28.19%), open abdominal surgeries (30.10%), and CDIV complications (153.07%).

Our findings suggest insurance type plays a significant role in outcomes and costs after colorectal surgery. Worse outcomes in Medicaid/Uninsured, low-SES patients were driven by increased presentation acuity, measured by increased odds of presenting with PASC and undergoing urgent/emergent surgeries, driving the increased complications and costs, consistent with a previous publication.^19^ Healthcare providers are increasingly being held accountable for quality, outcomes, and cost of care.^52,53^ Decreasing rates of nonelective surgeries is a potential target for policy change. Medicaid expansion was associated with a 1.8-percentage point increase in the probability of an early, uncomplicated presentation for several surgical conditions compared to states that did not expand Medicaid.^54^ Improving access to health care to decrease the incidence of PASC and urgent/emergent operations may be a better approach to improving surgical outcomes and reducing costs than P4P programs. A Cochrane review^55^ concluded that hospital P4P programs have an uncertain impact and effects on patient outcomes were at most small on quality of care, equity, or resource use. Further studies should assess the impact of improved healthcare access for vulnerable patients on reducing urgent/emergent surgeries, complications, and costs. Numerous studies indicate that SNH and academic medical centers are disproportionately penalized in current value-based medicine programs.^6,8,18^ Integration of socioeconomic risk factors into evaluation of high- and low-burden hospitals could improve the distribution/allocation of resources, improving resources to SNH and mitigating disparities potentially propagated by P4P programs.

### Limitations

This study is a retrospective review and cannot establish causal relationships. NSQIP provides a representative sample of surgeries but does not include all procedures performed at our institution. High complication and mortality rates may occur in cases performed for palliation, rather than for the purpose of extending life, but these data do not clearly define procedures that were performed specifically for palliation. While we included frailty, laparoscopic procedures and case status variables, multiple other variables could have been included.

## CONCLUSIONS

Medicaid/Uninsured insurance type was associated with decreased odds of achieving TO and increased odds of presenting with PASC and urgent/emergent cases, driving higher odds of complications, and index hospitalization costs. This suggests that factors beyond the surgeons’ control, such as increased presentation acuity and insurance type, impact surgical outcomes. Socioeconomic factors profoundly affect patient outcomes, which can account for the higher complication rates^47^ and costs of SNH serving a higher proportion of low-SES patients. Improving access to health care could provide a more significant impact on patient outcomes and decrease index hospitalization costs, by decreasing the incidence of urgent/emergent colorectal surgeries, particularly in low-SES, vulnerable patients.

## Acknowledgments

J.C.T. and M.A.J. are co-first authors and contributed equally to the contents of this study. P.K.S., J.K., M.A.J., and L.S.M. had full access to the data and take responsibility for the integrity of the data and the accuracy of the data analysis. Concept and design: P.K.S., S.S., M.A.J., J.K., B.B.B., and P.D. Acquisition, analysis, or interpretation of data: All authors. Drafting of the manuscript: J.C.T., M.A.J., and P.K.S. Critical revision of the manuscript for important intellectual content: All authors. Statistical analysis: M.A.J., J.K., C.-P.W., and P.D. Obtained funding: P.K.S., B.B.B., S.S., P.D., and C.-P.W. Administrative, technical, or material support: All authors. Supervision: P.K.S.

## Supplementary Material


