# Supplementary material for: Association of Insurance Type With Colorectal Surgery Outcomes and Costs at a Safety-Net Hospital: A Retrospective Observational Study
Source: Ann Surg Open. 2022 Nov 7;3(4):e215. doi: 10.1097/AS9.0000000000000215 (PMC9780053; doi:10.1097/AS9.0000000000000215)
Supplement: Supplementary file 1 [file as9-3-e215-s001.docx]

**Supplemental Digital Content:**

**SDC Table 1.** Distribution of PASC and CDIV Variables

**SDC Table 2.** Principal CPT Codes Used to Identify Colorectal Surgery Type

**SDC Table 3.** Distribution of Cases in the Medicaid/Uninsured Insurance Group

**SDC Table 4.** Any Complication and Outcome Variables used in Textbook Outcomes Adjusted for Frailty, Open Abdomen Procedures and Insurance Type for the Elective Cases Subgroup (N=620)

**SDC Table 5.** Any Complication and Outcome Variables used in Textbook Outcomes Adjusted for Frailty, Open Abdomen Procedures and Insurance Type for the Urgent/Emergent Cases Subgroup (N=458)

**SDC Table 6**. Length of Stay Adjusted for RAI, Open Abdomen With and Without Urgent/Emergent Case Status

**SDC Table 7.** Variable Costs for the No Postoperative Complications Subgroup Adjusted for RAI, Open Abdomen and Insurance

**SDC Table 8.** Variable Costs for the Textbook Outcomes Subgroup Adjusted for RAI, Open Abdomen and Insurance

**SDC Table 1. Distribution of PASC and CDIV Variables**

|  | **Overall** | **Private** | **Medicare** | **Medicaid/Uninsured** | **p-value** |
| --- | --- | --- | --- | --- | --- |
| **Preoperative Acute Serious Conditions (PASC)** | 149 | 19 | 32 | 98 |  |
| Deep incision surgical site infection (DSSIPATOS), No. (%) | 2 (1.3) | 1 (5.3) | 0 (0.0) | 1 (1.0) | .257 |
| Organ/space surgical site infection (OSSIPATOS), No. (%) | 40 (26.8) | 3 (15.8) | 7 (21.9) | 30 (30.6) | .318 |
| Pneumonia (PNAPATOS), No. (%) | 12 (8.1) | 2 (10.5) | 5 (15.6) | 5 (5.1) | .151 |
| On ventilator >48hrs (VENTPATOS), No. (%) | 13 (8.7) | 1 (5.3) | 5 (15.6) | 7 (7.1) | .285 |
| Sepsis (SEPSISPATOS), No. (%) | 81 (54.4) | 13 (68.4) | 11 (34.4) | 57 (58.2) | **.027** |
| Septic shock (SEPSHOCKPATOS), No. (%) | 40 (26.8) | 5 (26.3) | 12 (37.5) | 23 (23.5) | .298 |
| Acute renal failure not requiring dialysis^a^, No. (%) | 8 (5.4) | 0 (0.0) | 4 (12.5) | 4 (4.1) | .100 |
| Acute renal failure requiring dialysis^b^, No. (%) | 8 (5.4) | 1 (5.3) | 2 (6.2) | 5 (5.1) | .969 |
| Composite Distribution of PASC |  |  |  |  | .954 |
| Patients with 1 PASC, No. (%) | 106 (71.1) | 13 (68.4) | 21 (65.6) | 72 (73.5) |  |
| Patients with 2 PASC, No. (%) | 35 (23.5) | 5 (26.3) | 9 (28.1) | 21 (21.4) |  |
| Patients with 3 PASC, No. (%) | 5 (3.4) | 1 (5.3) | 1 (3.1) | 3 (3.1) |  |
| Patients with ≥4 PASC, No. (%) | 3 (2.0) | 0 (0.0) | 1 (3.1) | 2 (2.0) |  |
| Urgent/Emergent case status, No. (%) | 141 (94.6) | 16 (84.2) | 32 (100.0) | 93 (94.9) | .053 |
| **CDIV Complications** | 113 | 13 | 36 | 64 |  |
| Reintubation (REINTUB), No. (%) | 32 (28.3) | 7 (53.8) | 9 (25.0) | 16 (25.0) | .095 |
| Pulmonary embolism (PULEMBOL), No. (%) | 10 (8.8) | 2 (15.4) | 1 (2.8) | 7 (10.9) | .262 |
| Failure to wean (ventilator >48hrs) (FAILWEAN), No. (%) | 82 (72.6) | 11 (84.6) | 26 (72.2) | 45 (70.3) | .573 |
| Acute renal failure (require dialysis) (OPRENAFL), No. (%) | 6 (5.3) | 0 (0.0) | 1 (2.8) | 5 (7.8) | .371 |
| Cerebral vascular event/stroke (CNSCVA), No. (%) | 2 (1.8) | 0 (0.0) | 0 (0.0) | 2 (3.1) | .459 |
| Cardiac arrest (CDARREST), No. (%) | 10 (8.8) | 2 (15.4) | 5 (13.9) | 3 (4.7) | .202 |
| Myocardial infarction (CDMI), No. (%) | 17 (15.0) | 3 (23.1) | 9 (25.0) | 5 (7.8) | **.048** |
| Septic shock (OTHSESHOCK), No. (%) | 27 (23.9) | 3 (23.1) | 13 (36.1) | 11 (17.2) | .103 |
| Composite Distribution of CDIV complications |  |  |  |  | .077 |
| Patients with 1 CDIV | 69 (61.1) | 5 (38.5) | 20 (55.6) | 44 (68.8) |  |
| Patients with 2 CDIV | 23 (20.4) | 2 (15.4) | 9 (25.0) | 12 (18.8) |  |
| Patients with 3 CDIV | 16 (14.2) | 5 (38.5) | 4 (11.1) | 7 (10.9) |  |
| Patients with ≥4 CDIV | 5 (4.4) | 1 (7.7) | 3 (8.3) | 1 (1.6) |  |
| Urgent/Emergent case status | 88 (77.9) | 11 (84.6) | 26 (72.2) | 51 (79.7) | .568 |
| **Patients with PASC and CDIV** | 58 | 6 | 18 | 34 |  |
| Urgent/Emergent case status, No. (%) | 56 (96.6) | 5 (83.3) | 18 (100.0) | 33 (97.1) | .148 |

National Surgical Quality Improvement Program variable names included in parentheses

Pearson’s Chi-square tests used to calculate P values.

^a^defined as a positive response to RENAFAIL and negative response to DIALYSIS NSQIP variables

^b^defined as positive responses to both RENAFAIL and DIALYSIS NSQIP variables

Abbreviations: CDIV, Clavien-Dindo level IV complications; PASC, Preoperative Acute Serious Conditions; PATOS, present at time of surgery

| **SDC Table 2. Principal CPT Codes Used to Identify Colorectal Surgery Type**   \| **INCLUDED** \| \| **EXCLUDED** \| \| \| --- \| --- \| --- \| --- \| \| **Laparoscopic Procedures** \| **Open Abdominal Procedures*** \| **Perineal Procedures** \| **Transsacral Procedures** \| \| 44204 44205 44206 44207 44208 44210 44211 44212 45395 45397 45402 \| 44140 44141 44143 44144 44145 44146 44147 44150 44151 44155 44156 44157 44158 44160 45110 45111 45112 45113 45114 45119 45120 45121 45126 45135 45550 \| 45123  45130 \| 45116  45160 \| |
| --- | --- | --- | --- | --- | --- | --- | --- | --- | --- | --- | --- | --- |
| CPT, Current Procedural Terminology  *Includes combined open abdominal and perineal procedures |

**SDC Table 3. Distribution of Cases in the Medicaid/Uninsured Insurance Group**

| **Medicaid/Uninsured Insurance Total, No** | **619** |
| --- | --- |
| County Indigent Programs, No. (%) | 281 (45.4) |
| Charity care, No. (%) | 8 (1.3) |
| Medicare/Medicaid Dual Eligible, No. (%) | 70 (11.3) |
| Medicaid, No. (%) | 161 (26.0) |
| Self-pay <1% charges collected, No. (%) | 99 (16.0) |

**SDC Table 4. Any Complication and Outcome Variables used in Textbook Outcomes Adjusted for Frailty, Open Abdomen Procedures and Insurance Type for the Elective Cases Subgroup (N=620)**

|  | **Any Complication** | | | | **CDIV Complications** | | | | **Reoperations** | | |
| --- | --- | --- | --- | --- | --- | --- | --- | --- | --- | --- | --- |
|  | aOR | | CI | p-value | aOR | CI | p-value | | aOR | CI | p-value |
| **RAI (Ref = Normal 21-29)** | | | |  |  |  |  | |  |  |  |
| Robust (≤20) | 1.16 | | 0.75 – 1.81 | .515 | 1.02 | 0.38 – 2.92 | .966 | | 1.01 | 0.47 – 2.30 | .983 |
| Frail (30-39) | 1.63 | | 0.80 – 3.34 | .179 | 0.69 | 0.10 – 2.99 | .653 | | 1.15 | 0.30 – 3.63 | .819 |
| Very Frail (≥40) | 8.12 | | 1.28 – 158.83 | .060 | 6.42 | 0.79 – 38.26 | **.049** | | 1.87 | 0.09 – 12.63 | .579 |
| **Open Abdomen** | 3.71 | | 2.60 – 5.34 | **<.001** | 2.27 | 0.96 – 5.82 | .070 | | 1.69 | 0.89 – 3.26 | .111 |
| **(Ref = Laparoscopic)** | | | |  |  |  |  | |  |  |  |
| **Insurance (Ref = Private)** | | |  |  |  |  |  |  | |  |  |
| Medicare | 1.52 | | 0.87 – 2.66 | .143 | 7.30 | 1.68 – 51.14 | **.017** | | 1.08 | 0.39 – 2.93 | .885 |
| Medicaid/Uninsured | 1.25 | | 0.83 – 1.91 | .293 | 3.47 | 0.93 – 22.46 | .107 | | 1.16 | 0.56 – 2.53 | .699 |
|  | **30-day EDOS** | | | | **30-day Readmissions** | | | |  |  |  |
|  | aOR | CI | | p-value | aOR | CI | p-value | |  |  |  |
| **RAI (Ref = Normal 21-29)** | |  | | | | |  | |  |  |  |
| Robust (≤20) | 1.47 | 0.78 – 2.94 | | .254 | 1.14 | 0.68 – 1.96 | .617 | |  |  |  |
| Frail (30-39) | 2.44 | 0.92 – 6.22 | | .065 | 0.96 | 0.40 – 2.15 | .924 | |  |  |  |
| Very Frail (≥40) | 4.00 | 0.51 – 22.39 | | .133 | 2.41 | 0.44 – 11.83 | .275 | |  |  |  |
| **Open Abdomen** | 1.24 | 0.75 – 2.06 | | .397 | 2.94 | 1.91 – 4.59 | **<.001** | |  |  |  |
| **(Ref = Laparoscopic)** | | | |  |  |  |  | |  |  |  |
| **Insurance (Ref = Private)** | |  | | | | |  | |  |  |  |
| Medicare | 2.09 | 0.74 – 6.19 | | .169 | 1.46 | 0.74 – 2.88 | .270 | |  |  |  |
| Medicaid/Uninsured | 5.77 | 2.74 – 14.16 | | **<.001** | 1.46 | 0.89 – 2.47 | .142 | |  |  |  |

Abbreviations: aOR, Adjusted Odds Ratio; CI, Confidence Interval; PASC, Preoperative Acute Serious Conditions; RAI, Risk Analysis Index

30-day EDOS and Readmissions defined as 30 days from date of discharge from the index hospitalization

30-day Mortality not reported due to no patients with Private insurance dying within 30 days of surgery, model estimates not reported.

|  | **Any Complication** | | | **CDIV Complications** | | | **Reoperations** | | |
| --- | --- | --- | --- | --- | --- | --- | --- | --- | --- |
|  | aOR | CI | p-value | aOR | CI | p-value | aOR | CI | p-value |
| **RAI (Ref = Normal 21-29)** | | |  |  |  |  |  |  |  |
| Robust (≤20) | 0.45 | 0.28 – 0.71 | **<.001** | 0.34 | 0.19 – 0.62 | **<.001** | 1.28 | 0.60 – 2.89 | .540 |
| Frail (30-39) | 1.36 | 0.70 – 2.71 | .379 | 1.14 | 0.57 – 2.26 | .699 | 1.40 | 0.51 – 3.64 | .494 |
| Very Frail (≥40) | 1.81 | 0.61 – 6.71 | .321 | 1.42 | 0.51 – 3.79 | .493 | 0.42 | 0.02 – 2.39 | .426 |
| **Open Abdomen** | 3.26 | 2.06 – 5.26 | **<.001** | 3.91 | 1.90 – 9.16 | **<.001** | 2.86 | 1.19 – 8.50 | **.032** |
| **(Ref = Laparoscopic)** | | |  |  |  |  |  |  |  |
| **Insurance (Ref = Private)** | |  |  |  |  |  |  |  |  |
| Medicare | 2.12 | 1.04 – 4.38 | **.040** | 1.95 | 0.85 – 4.68 | .124 | 2.66 | 0.85 – 9.35 | .104 |
| Medicaid/Uninsured | 1.89 | 1.11 – 3.25 | **.020** | 1.24 | 0.62 – 2.69 | .559 | 1.51 | 0.61 – 4.58 | .417 |
|  | **30-day Mortality** | | | **30-day EDOS** | | | **30-day Readmissions** | | |
|  | aOR | CI | p-value | aOR | CI | p-value | aOR | CI | p-value |
| **RAI (Ref = Normal 21-29)** | |  | | | |  |  |  |  |
| Robust (≤20) | 0.26 | 0.07 – 0.86 | **.032** | 0.77 | 0.40 – 1.49 | .423 | 0.85 | 0.50 – 1.45 | .546 |
| Frail (30-39) | 1.71 | 0.53 – 5.21 | .346 | 0.42 | 0.12 – 1.19 | .132 | 1.25 | 0.61 – 2.53 | .534 |
| Very Frail (≥40) | 4.55 | 1.23 – 15.80 | **.018** | 0.63 | 0.09 – 2.46 | .556 | 1.75 | 0.60 – 4.77 | .282 |
| **Open Abdomen** | 2.98 | 0.83 – 19.11 | .151 | 1.36 | 0.69 – 2.88 | .396 | 1.57 | 0.92 – 2.81 | .111 |
| **(Ref = Laparoscopic)** | | |  |  |  |  |  |  |  |
| **Insurance (Ref = Private)** | |  | | | |  |  |  |  |
| Medicare | 0.99 | 0.23 – 5.10 | .990 | 1.93 | 0.54 – 7.86 | .324 | 1.22 | 0.51 – 3.02 | .656 |
| Medicaid/Uninsured | 1.14 | 0.35 – 5.19 | .841 | 3.13 | 1.21 – 10.67 | **.035** | 2.09 | 1.08 – 4.38 | **.037** |

**SDC Table 5. Any Complication and Outcome Variables used in Textbook Outcomes Adjusted for Frailty, Open Abdomen Procedures and Insurance Type for the Urgent/Emergent Cases Subgroup (N=458)**

Abbreviations: aOR, Adjusted Odds Ratio; CI, Confidence Interval; PASC, Preoperative Acute Serious Conditions; RAI, Risk Analysis Index

30-day EDOS and Readmissions defined as 30 days from date of discharge from the index hospitalization

30-day Mortality defined as 30 days from date of index surgery

**SDC Table 6. Length of Stay Adjusted for RAI, Open Abdomen With and Without Urgent/Emergent Case Status**

|  | **Log(Length of Stay)** | | | | **Log(Length of Stay)** | | | |
| --- | --- | --- | --- | --- | --- | --- | --- | --- |
|  | %change | Estimates | CI | p-value | %change | Estimates | CI | p-value |
| **RAI (Ref = Normal 21-29)** | |  |  |  |  |  |  |  |
| Robust (≤20) | -17.18 | -0.188 | -0.282, -0.095 | **< .001** | -12.42 | -0.133 | -0.219, -0.046 | **.003** |
| Frail (30-39) | 17.30 | 0.160 | 0.016, 0.303 | **.029** | 12.67 | 0.119 | -0.013, 0.252 | .077 |
| Very Frail (≥40) | 31.95 | 0.277 | 0.024, 0.531 | **.032** | 18.81 | 0.172 | -0.062, 0.407 | .149 |
| **Open Abdomen**  **(Ref = Laparoscopic)** | 65.21 | 0.502 | 0.422, 0.582 | **< .001** | 45.37 | 0.374 | 0.299, 0.450 | **< .001** |
| **Insurance (Ref = Private)** | |  |  |  |  |  |  |  |
| Medicare | 6.06 | 0.059 | -0.068, 0.185 | .362 | 6.69 | 0.065 | -0.052, 0.181 | .276 |
| Medicaid/Uninsured | 17.51 | 0.161 | 0.067, 0.256 | **< .001** | 8.74 | 0.084 | -0.004, 0.171 | .061 |
| **Urgent/Emergent (Ref = Elective)** | | | |  | 70.42 | 0.533 | 0.457, 0.609 | **< .001** |

Abbreviations: aOR, Adjusted Odds Ratio; CI, Confidence Interval; RAI, Risk Analysis Index; Ref, Reference Value

**SDC Table 7. Variable Costs for the No Postoperative Complications Subgroup Adjusted for RAI, Open Abdomen and Insurance**

|  | **Log(variable costs)** | | | | **Log(variable costs)** | | | |
| --- | --- | --- | --- | --- | --- | --- | --- | --- |
|  | %change | Estimates | CI | p-value | %change | Estimates | CI | p-value |
| Intercept |  | 9.03 | 8.94 – 9.13 | **<.001** |  | 8.98 | 8.89 – 9.08 | **<.001** |
| **RAI (Ref = Normal 21-29)** | |  |  |  |  |  |  |  |
| Robust (≤20) | -11.21 | -0.12 | -0.20 – -0.04 | **.004** | -10.87 | -0.12 | -0.19 – -0.04 | **.003** |
| Frail (30-39) | 13.07 | 0.12 | -0.02 – 0.26 | .083 | 10.98 | 0.10 | -0.03 – 0.24 | .122 |
| Very Frail (≥40) | 29.11 | 0.26 | -0.09 – 0.60 | .147 | 14.72 | 0.14 | -0.19 – 0.47 | .414 |
| **Open Abdomen**  **(Ref = Laparoscopic)** | 15.13 | 0.14 | 0.08 – 0.20 | **<.001** | 8.23 | 0.08 | 0.02 – 0.14 | **.011** |
| **Insurance (Ref = Private)** | |  |  |  |  |  |  |  |
| Medicare | 1.47 | 0.01 | -0.09 – 0.12 | .789 | 2.40 | 0.02 | -0.08 – 0.13 | .649 |
| Medicaid/Uninsured | 8.84 | 0.08 | 0.01 – 0.16 | **.022** | 5.60 | 0.05 | -0.01 – 0.12 | .123 |
| **Urgent/Emergent (Ref = Elective)** | | | |  | 29.84 | 0.26 | 0.20 – 0.33 | **<.001** |

Abbreviations: CI, Confidence Interval; RAI, Risk Analysis Index; Ref, Reference Value;

Note: % change is calculated with marginal change of Log(direct costs) for one unit of each variable change below

($e$^(intercept+estimated coefficients)^ -$e$^intercept^ )/$e$^intercept^ *100, which is equal to ($e$^estimated coefficients^ -1)*100

**SDC Table 8. Variable Costs for the Textbook Outcomes Subgroup Adjusted for RAI, Open Abdomen and Insurance**

|  | **Log(variable costs)** | | | | **Log(variable costs)** | | | |
| --- | --- | --- | --- | --- | --- | --- | --- | --- |
|  | %change | Estimates | CI | p-value | %change | Estimates | CI | p-value |
| Intercept |  | 9.03 | 8.92 – 9.13 | **<.001** |  | 8.97 | 8.87 – 9.07 | **<.001** |
| **RAI (Ref = Normal 21-29)** | |  |  |  |  |  |  |  |
| Robust (≤20) | -10.62 | -0.11 | -0.20 – -0.03 | **.011** | -9.34 | -0.10 | -0.18 – -0.01 | **.022** |
| Frail (30-39) | 21.13 | 0.19 | 0.05 – 0.33 | **.008** | 18.12 | 0.17 | 0.03 – 0.30 | **.017** |
| Very Frail (≥40) | 16.16 | 0.15 | -0.31 – 0.61 | .522 | 4.92 | 0.05 | -0.39 – 0.49 | .831 |
| **Open Abdomen**  **(Ref = Laparoscopic)** | 32.72 | 0.28 | 0.21 – 0.35 | **<.001** | 24.77 | 0.22 | 0.15 – 0.29 | **<.001** |
| **Insurance (Ref = Private)** | |  |  |  |  |  |  |  |
| Medicare | 2.55 | 0.03 | -0.09 – 0.14 | .665 | 3.57 | 0.04 | -0.08 – 0.15 | .532 |
| Medicaid/Uninsured | 13.48 | 0.13 | 0.04 – 0.21 | **.002** | 9.58 | 0.09 | 0.01 – 0.17 | **.024** |
| **Urgent/Emergent (Ref = Elective)** | | | |  | 31.17 | 0.27 | 0.20 – 0.34 | **<.001** |

Abbreviations: CI, Confidence Interval; RAI, Risk Analysis Index; Ref, Reference Value;

Note: % change is calculated with marginal change of Log(direct costs) for one unit of each variable change below

($e$^(intercept+estimated coefficients)^ -$e$^intercept^ )/$e$^intercept^ *100, which is equal to ($e$^estimated coefficients^ -1)*100
